# Niche and interspecific associations of dominant tree species in *Castanopsis eyrei* and *Castanopsis carlesii* communities in Meihua Mountain, Fujian

**DOI:** 10.3389/fpls.2025.1703968

**Published:** 2026-01-27

**Authors:** Jiali Yu, Mengwei Chi, Chenyu Gong, Menglin Chang, Xing He, Shipin Chen, Jinping Wu, Liang Ma, Siren Lan

**Affiliations:** 1 College of Forestry, Fujian Agriculture and Forestry University, Fuzhou, China; 2 College of Landscape Architecture and Art, Fujian Agriculture and Forestry University, Fuzhou, China; 3 Bureau of Meihua Mountain National Nature Reserve, Longyan, China; 4 Fujian Health College, Fuzhou, China

**Keywords:** evergreen broad-leaved forest, interspecies association, Meihua Mountain nature reserve, niche, woody plant

## Abstract

**Introduction:**

Species composition, interspecific associations, and community stability play crucial roles in shaping individual plant survival and population dynamics. Research in this area carries multidimensional significance for forest conservation, contributing to the maintenance of ecological balance and the enhancement of biodiversity. To explore interspecific interactions among dominant species in evergreen broad-leaved forest communities and promote favorable community development, we selected two representative communities dominated by *Castanopsis eyrei* and *Castanopsis carlesii* for detailed investigation.

**Methods:**

Using methods such as niche analysis, variance ratio (*VR*), chi-square test (*χ*²), and Spearman’s rank correlation, we analyzed the niche characteristics and interspecific association patterns of the 13 tree species with the highest importance values (*IV*) in each community.

**Results:**

Our results revealed high species richness, with *Castanopsis carlesii* exhibiting the highest importance value and a relatively wide niche breadth, confirming its dominant role. However, the ranking of niche breadth did not correspond directly to the importance value ranking, suggesting that species distribution frequency plays a key role in determining niche width. The average niche overlap (0.26) and niche similarity coefficient (0.29) among dominant species were low, indicating limited resource sharing. Overall, interspecific associations showed a non-significant negative trend, both *χ*² and Spearman’s tests positive-to-negative association ratio was 0.77.

**Discussion:**

Contrary to previous studies that suggest relative stability in evergreen broad-leaved forests, our findings indicate that the studied communities are currently in a relatively unstable developmental stage. This instability highlights the need for strategic adjustments in species composition and enhanced promotion of positive interspecific relationships. We therefore recommend deliberate optimization of tree species assemblages to strengthen facilitative interactions and improve community resilience.

## 1 Introduction

In plant communities, there are intricate interspecific relationships, including symbiosis, competition, and co-evolution, and these relationships are centered on the characteristics of niches and interspecies association (Graff et al., 2007; Li et al., 2019; Su et al., 2015). Community ecology studies are founded on understanding the processes that sustain long-term species coexistence under spatially constrained resource conditions (Bengtsson et al., 1994). Niche differentiation resulting from interspecific interactions is a key factor in species coexistence (Holt et al., 1987). It is possible to determine the coupling between a species and its environment by looking at its ecological niches, which provide insight into how species cohabit and represent their potential to use resources (Deng et al., 2024). Niche characteristics are primarily composed of niche width, niche overlap, and niche similarity (Pastore et al., 2021). Niche width reflects a species’ environmental adaptability, with broader niches indicating greater resource utilization efficiency (Xu et al., 2021). Niche overlap, which quantifies the similarity in resource use among species, demonstrates that taxa with higher overlap exhibits more comparable resource exploitation strategies (Pastore et al., 2021). Understanding species’ resource-use traits, coexistence strategies, community stability, and composition is enabled by interspecies associations, which also reveal trends in community succession and species composition (Jin et al., 2022).

Niche and interspecific associations comprehensively reveal species interactions, fundamental community structures, and successional dynamics—critical theoretical and practical contributions to natural vegetation restoration and biodiversity conservation. For instance, interspecific interactions are intimately related to the stability of forest stand structure, with positive correlations being particularly critical and contributing positively to the stability and development of forest community structure in Vietnam (Nguyen et al., 2023). According to Pandey et al. (2023), species composition is crucial in India’s Himalayan area, and species survival is significantly impacted by the niche and breadth of species. Zhang et al. (2024) found that vegetation in the early stages of succession tends to exhibit relatively independent population dynamics. In addition, the vegetation community structure of ecosystems is also influenced by tree species diversity, anthropogenic disturbances, and altitudinal shifts (Zhao et al., 2024; Bhandari et al., 2000).

Niche and interspecies associations have emerged as hot topics of global discussion, garnering significant scholarly attention. Cornell et al. (1984) emphasize that there is a dynamic relationship between niche and plant diversity, with species diversity reaching its maximum under moderately disturbed environmental conditions. Clause (1934) argued that when two species concurrently exploit identical limited resources, one species gains competitive dominance while the other faces exclusion. Gidudu et al. (2011) concluded that habitat heterogeneity shapes interspecific interactions, with positive associations more probable among species occupying comparable niches. Luo et al.’s (2012) examination of tree species spatial distributions in subtropical broad-leaved evergreen forests suggests that interspecific positive associations may influence forest species assemblages. These studies offer a theoretical foundation for comprehending interspecific associations features and ecological niches.

Understory vegetation is a driver of forest ecosystems and plays an important role in maintaining community balance and regulating the ecological structure of forest trees (Nilsson and Wardle, 2005). The composition of understory vegetation in Fujian Province is rich (Farooq et al., 2019). Meihua Mountain Nature Reserve is in an area of extensive broad-leaved evergreen forests, which is a key land area with significant biodiversity. *Castanopsis carlesii* and *Castanopsis eyrei* are evergreen tree species belonging to the genus *Castanopsis* (Fagaceae). They exhibit strong adaptability to various habitats and serve as dominant or constructive species in subtropical evergreen broad-leaved forests, playing a crucial role in maintaining community stability (Lin et al., 2017). *Schima superba* is also a common evergreen broad-leaved plant in subtropical forests of China (Gilani et al., 2025). However, due to large-scale human disturbances, such as artificial logging operations, a significant portion of the once pristine primary forest in this area has undergone secondary succession. This ecological change has disrupted the original forest structure and composition, making it imperative to conduct an in-depth analysis of the community succession patterns. Although numerous scholars have conducted foundational research on the population structure, species composition, and community assembly of evergreen broad-leaved forests in surrounding regions in recent years (Kong and Li, 2012; Chen, 2006), research on the evergreen broad-leaved forest communities in Meihua Mountain Nature Reserve remains limited, particularly regarding niche differentiation and interspecific associations within these communities. Furthermore, previously, while studies on niche and interspecific associations have been conducted in other communities at Meihua mountain, these investigations have only focused on either the niche or interspecific association individually. They have failed to organically integrate these two aspects, nor have they clarified the intrinsic relationship between niche and interspecific association, as well as their connection with species coexistence mechanisms. This knowledge gap has resulted in an insufficient comprehensive and in-depth understanding of the symbiotic mechanisms within the evergreen broad-leaved forest communities at Meihua mountain.

This research focuses on the evergreen broad - leaved forest communities within forest ecosystems. The aim is to mitigate both intra - specific and interspecific competition and strengthen synergistic symbiotic relationships through a profound analysis of the niche characteristics of dominant species and their interspecific interactions. Ultimately, this will enhance the stability of the ecosystem and offer a scientific foundation for forest and biodiversity conservation. To attain this objective, we utilized the methodologies of niche and interspecific association to tackle the following problems:(1) determine the niche breadths and overlap patterns of dominant species populations within communities, revealing resource utilization strategies and interspecific competitive intensities; (2) clarify the positive and negative association patterns between dominant tree species pairs, thereby optimizing vegetation configuration to achieve community stability; (3) elucidate the mechanistic basis of resource partitioning in shaping interspecific interactions through coupled analysis of niche overlap indices and interspecific correlation coefficients. These studies can provide a comprehensive understanding of the succession mechanisms of evergreen broad-leaved forest communities, which is of great significance for the protection of ecosystems, the maintenance of biodiversity, and sustainable development.

## 2 Materials and methods

### 2.1 Study area

The Meihua Mountain Nature Reserve is located at the intersection of Shanghang County, Liancheng County, and Xinluo District in Fujian Province, China (25°15’14’’–25°35’44’’N, 116°45’25’’–116°57’33’’E), covering a total area of 22,168.5 hectares. The reserve is characterized by medium-elevation mountainous terrain, with a topographic configuration marked by higher elevations in the central and western regions and lower elevations toward the periphery and east, averaging approximately 900 meters above sea level. Climatically, the reserve lies within the transitional zone between the southern edge of the mid-subtropics and the northern part of the south subtropics. Due to its proximity to the coast, it is regularly influenced by warm and humid southeasterly maritime air masses, resulting in a prolonged rainy season, abundant precipitation, and frequent heavy rainfall events. Annual precipitation ranges from 1,700 to 2,200 millimeters, and relative humidity remains consistently high, varying between 70% and 96%. The area is recognized as one of the major storm-prone regions in Fujian Province. The dominant soil type is red soil, and the zonal vegetation consists primarily of evergreen broad-leaved forest. Major vegetation formations include those dominated by *Castanopsis eyrei*, *Castanopsis carlesii*, *Schima superba*, and *Machilus thunbergii*.

### 2.2 Sample survey

**Figure 1:**
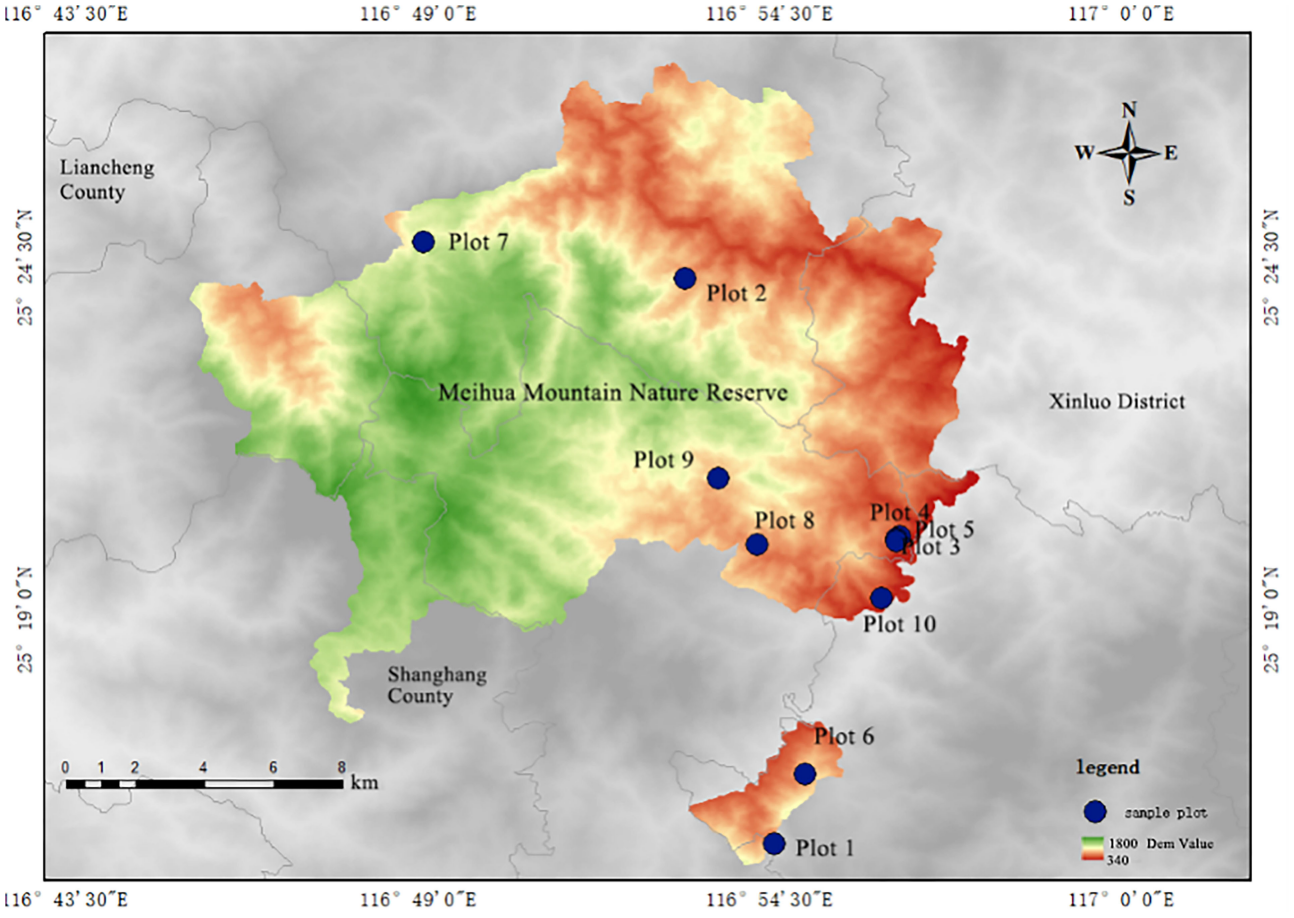
Distribution map of sample plots and research flowchart. **(a)** Research Area - Fujian, **(b)** Research Area - Meihuashan Nature Reserve, Fujian **(c)** Research Flowchart.

Between December 2023 to August 2024, a comprehensive field investigation was conducted in the nature reserve during the period of vigorous plant growth, avoiding extreme weather conditions and anthropogenic disturbances. The study applied the standard plot method, using forest formations as sampling units to develop a formation classification system. Ten main plots, each measuring 20 m × 20 m, were established within spatially representative formations (primarily regions dominated by *Castanopsis eyrei* and *Castanopsis carlesii*). Each main plot was systematically subdivided into four 10 m × 10 m subplots (**Figure 1**). Each subplot was demarcated with boundary stakes and delineated using flagging tape. Woody plants with a diameter at breast height (DBH) ≥5.0 cm were classified as members of the tree layer; individual tree data—including species identity, DBH, height, basal diameter, and crown width—were recorded *in situ*. Tree height was measured using an altimeter (accuracy ±0.1 cm), while basal diameter and crown width were recorded with a steel tape measure (accuracy ±0.1 cm), and DBH was measured using digital calipers (accuracy ±0.1 cm). Geographic attributes for each subplot, including local place name, elevation, slope gradient, and slope aspect, were documented (**Table 1**). Canopy density was estimated using the grid-point intercept method, calculated as the ratio of crown projection points to total grid points. The selected plots span a significant elevational gradient and are broadly distributed across the landscape, resulting in notable variation in temperature, precipitation, and other abiotic factors. This spatial heterogeneity supports the development of diverse hydrothermal regimes across the study area.

**Table 1:** Detailed information on the survey sample plots.

Sample plot	Location	Altitude	Slope	Slope direction	Canopy density
1	116°54′34.20′′E 25°15′30.60′′N	706 m	Gentle slope	Southwest	85%
2	116°53′11.04′′E 25°24′20.52′′N	855 m	Gentle slope	North	86%
3	116°56′32.64′′E 25°20′18.24′′N	762 m	Steep slope	Southeast	80%
4	116°56′28.68′′E 25°20′16.44′′N	788 m	Gentle slope	Northeast	89%
5	116°56′29.4′′E 25°20′14.24′′N	815 m	Gentle slope	Southeast	85%
6	116°55′3.72′′E 25°16′35.76′′N	674 m	Gentle slope	West	85%
7	116°49′6.24′′E 25°24′54.00′′N	1093 m	Gentle slope	Southwest	83%
8	116°54′18.36′′E 25°20′10.68′′N	772 m	Steep slope	Northeast	85%
9	116°53′42.00′′E 25°21′12.96′′N	951 m	Gentle slope	Southeast	40%
10	116°56′50.28′′E 25°18′41.04′′N	518 m	Sharp slope	Southwest	70%

Gentle slopes (6–15 degrees), steep slopes (26–35 degrees), sharp slopes (36–45 degrees).

In this study, a total of 65 tree species from 38 genera and 27 families were recorded. Based on the plot survey data, the importance value (*IV*) was calculated for each species. Species were ranked according to their *IV_s_*, and those with an *IV* exceeding 1.5% were classified as dominant, following the method described by Deng et al. (2024). A total of 13 dominant tree species were identified, contributing a cumulative importance value of 71.32% and comprising 440 individuals, representing 69.03% of the total tree population. These species effectively represent the structural and compositional characteristics of the community and were therefore selected for subsequent analyses of niche differentiation and interspecific associations.

Niche breadth, niche overlap, and niche similarity were calculated for the dominant species to assess their environmental adaptability and resource utilization patterns. Overall interspecific association was assessed using the variance ratio (*VR*), while species coexistence mechanisms and competitive interactions were examined through statistical approaches, including the chi-square test (*χ*²) and Spearman’s rank correlation coefficient.

### 2.3 Statistic analysis

#### 2.3.1 Importance value

Importance value (Collins et al., 1895) is a comprehensive quantitative metric used to assess species dominance within a community. It was calculated using three components: relative density (*RD*), relative frequency (*RF*), and relative prominence (*RP*).

#### 2.3.2 Niche characteristics

Following the method of Pianka (1973), Levins index (*B_L_*, Equation 1) and Shannon-Wiener Index (*B_S_*, Equation 2) were calculated for each dominant species to assess niche breadth. Additionally, the Pianka index (*O_ik_*, Equation 3) and Schoener index (*C_ik_*, Equation 4) were computed to evaluate niche overlap and niche similarity, respectively (Pianka, 1974). Both indices range from 0 to 1, with higher *O_ik_* values indicating greater niche overlap and higher *C_ik_* values reflecting greater niche similarity.

(1) $$B_{L}=1/\sum_{j=1}^{r}P_{ij}^{2}$$

(2) $$B_{S}=-\sum_{i=1}^{r}P_{ij}lnP_{ij}$$

(3) $$O_{ik}=\sum_{j=1}^{r}P_{ij}P_{kj}/\sqrt{\sum_{j=1}^{r}P_{ij}^{2}\sum_{j=1}^{r}P_{kj}^{2}}$$

(4) $$C_{ik}=1-1/2\sum_{j=1}^{r}\left|P_{ij}-P_{kj}\right|$$

In the formula, *r* represents the number of quadrats, while *P_ij_* and *P_kj_* denote the proportions of the importance values of species *i* and species *k* in plot *j* relative to the total importance values across all quadrats, respectively. *O_ik_* represents the degree of niche overlap between species *i* and species *k*, ranging from 0 to 1. When *O_ik_* approaches 0, niche overlap between the two species is low; conversely, as *O_ik_* approaches 1, niche overlap increases.

#### 2.3.3 Overall interspecific association and interspecific association

To evaluate the overall interspecific association, a 2×2 contingency table was constructed based on the survey data. The variance ratio (Equation 5), following Schluter (1984) was used to assess the overall interspecific association among tree species in the community, and the test statistic *W* was used to determine the significance of the association. A *VR* value greater than 1 indicates a positive overall interspecific association, a value less than 1 indicates a negative association, and a value equal to 1 suggests no association. Statistic *W* evaluates the significance of the deviation of the observed *VR* from the expected null distribution. When $\chi_{0.95,N}^{2}$< *W<* $\chi_{0.05,N}^{2}$, the association is considered non-significant; otherwise, it is deemed significant. This analytical framework identifies significant associations among dominant species and facilitates simultaneous evaluation of interspecific association patterns across the community.

Based on the *χ*^2^ (Equation 6), this study conducts a comprehensive analysis of interspecific association by integrating the Spearman rank correlation coefficient (Zar, 1972). The degree of interspecific association was analyzed qualitatively using the *χ*² statistic based on the distribution data of the 13 dominant species in the sampling plots and a 2×2 contingency table. Considering the discontinuous sampling method, the *χ*² test was corrected using Yates’ continuity correction formula. Spearman rank correlation coefficient (Equation 7) provides quantitative analysis of species associations based on the numerical relationships between species in the plots. At a 0.05 significance level, this study employs these two correlation analysis methods to mutually validate the reliability of analytical results. This multi-method approach enables a comprehensive assessment of correlations between variables from different perspectives, thereby objectively and accurately reflecting the strength and significance of linear associations between species.

(5) $$VR=\frac{S_{T}^{2}}{Q_{T}^{2}}=\frac{\frac{1}{N}\sum_{j=1}^{N}{\left(T_{j}-t\right)}^{2}}{\sum_{i=1}^{S}\frac{n_{i}}{N}\left(1-\frac{n_{i}}{N}\right)}$$

In the formula, *S* represents the number of individuals to be tested; *N* denotes the total number of selected quadrats; *ni* indicates the frequency of occurrence of species *i* in each quadrat; *T_j_* represents the number of species in quadrat *j*; and *t* is the average number of species across all quadrats. The variance ratio method can be used to test the overall interspecific association among multiple species.

(6) $$\chi^{2}=N{\left(\left|ad-bc\right|\mathrm{\&-}\frac{N}{2}\right)}^{2}/\left(a+b\right)\left(a+c\right)\left(b+d\right)\left(c+d\right)$$

In the formula, *N* denotes the total number of sample quadrats; *a* denotes the number of quadrats in which both species co-occur; *b* and *c* denote the number of quadrats in which each species occurs independently; and *d* denotes the number of quadrats in which neither species is present. When *χ*²< 3.841, the interspecific association is not significant (*p* > 0.05); when 3.841 ≤ *χ*² ≤ 6.635, the association is significant (0.01< *p* ≤ 0.05); and when *χ*² > 6.635, the association is highly significant (*p* < 0.01).

(7) $$r_{S}\left(i,s\right)=1-\frac{6\sum_{j=1}^{N}{\left(x_{ij}-\bar{x_{i}}\right)}^{2}{\left(x_{kj}-\bar{x_{k}}\right)}^{2}}{N^{3}-N}$$

In the formula, *x_ij_* and *x_kj_* represent the number of individuals of species *i* and species *k* in plot *j*, respectively, with values ranging from -1 to 1. A positive result indicates a positive interspecific correlation, while a negative result indicates a negative interspecific correlation.

### 2.4 Software

Microsoft Excel 2010 was used for data calculation and organization. ArcGIS 10.8 generated the sample plot distribution map. Origin 2024 created the relationship diagram between importance value and niche breadth, heatmaps of niche overlap and similarity, and the heatmaps visualizing Spearman rank correlation coefficients. The rest of the graphs are generated by Excel.

## 3 Results

### 3.1 Importance value and niche breadth analysis

According to **Table 2**, *Castanopsis carlesii* exhibited the highest importance value (21.27%) among the 13 dominant species and functioned as the constructive species in the community. This was followed by *Castanopsis eyrei*, *Phyllostachys edulis*, and *Machilus thunbergii*, with importance values of 19.33%, 5.54%, and 4.17%, respectively, while *Castanopsis lamontii* had the lowest importance value at 1.63%. For the 13 dominant tree species, the mean Levins index (*B_L_*) and Shannon–Wiener index (*B_S_*) were 3.40 and 1.10, respectively, indicating a relatively differentiated pattern of resource utilization among these species. *Machilus thunbergii* displayed the largest niche breadth (*B_L_* = 5.72, *B_S_* = 1.82), reflecting its superior environmental adaptability and competitive ability. *Castanopsis carlesii* and *Castanopsis eyrei* ranked next in niche breadth (*B_L_* = 5.56, *B_S_* = 1.75 and *B_L_* = 5.36, *B_S_* = 1.72, respectively), suggesting that these two species can acquire resources and maintaining survival and development across diverse environmental conditions. In contrast, certain species such as *Castanopsis tibetana* exhibited a Shannon–Wiener index of 0.00, which may indicate highly specialized or restricted resource utilization within the community.

**Table 2:** Ecological niche characteristics of dominant species.

Species number	Species	Number of individuals	Importance value	Niche breadth	
				*B_L_*	*B_S_*
S1	*Castanopsis carlesii* (Hemsl.) Hayata	119	21.27%	5.56	1.75
S2	*Castanopsis eyrei* (Champ. ex Benth.) Tutcher	85	19.33%	5.36	1.72
S3	*Phyllostachys edulis* (Carrière) J. Houz.	80	5.54%	4.80	0.96
S4	*Machilus thunbergii* Siebold&Zucc.	25	4.17%	5.72	1.82
S5	*Rhododendron henryi* Hance	26	2.96%	2.87	1.08
S6	*Schima superba* Gardner&Champ.	10	2.91%	3.82	1.44
S7	*Myrsine seguinii* H. Lév.	19	2.87%	1.56	0.66
S8	*Castanopsis tibetana* Hance	16	2.41%	1.00	0.00
S9	*Sloanea sinensis* (Hance) Hemsl.	15	2.33%	1.97	0.69
S10	*Daphniphyllum oldhamii* (Hemsl.) K. Rosenth.	19	2.18%	2.04	0.86
S11	*Altingia gracilipes* Hemsl.	6	2.03%	2.00	0.69
S12	*Aidia cochinchinensis* Lour.	13	1.68%	4.79	1.59
S13	*Castanopsis lamontii* Hance	7	1.63%	2.75	1.05

**Figure 2:**
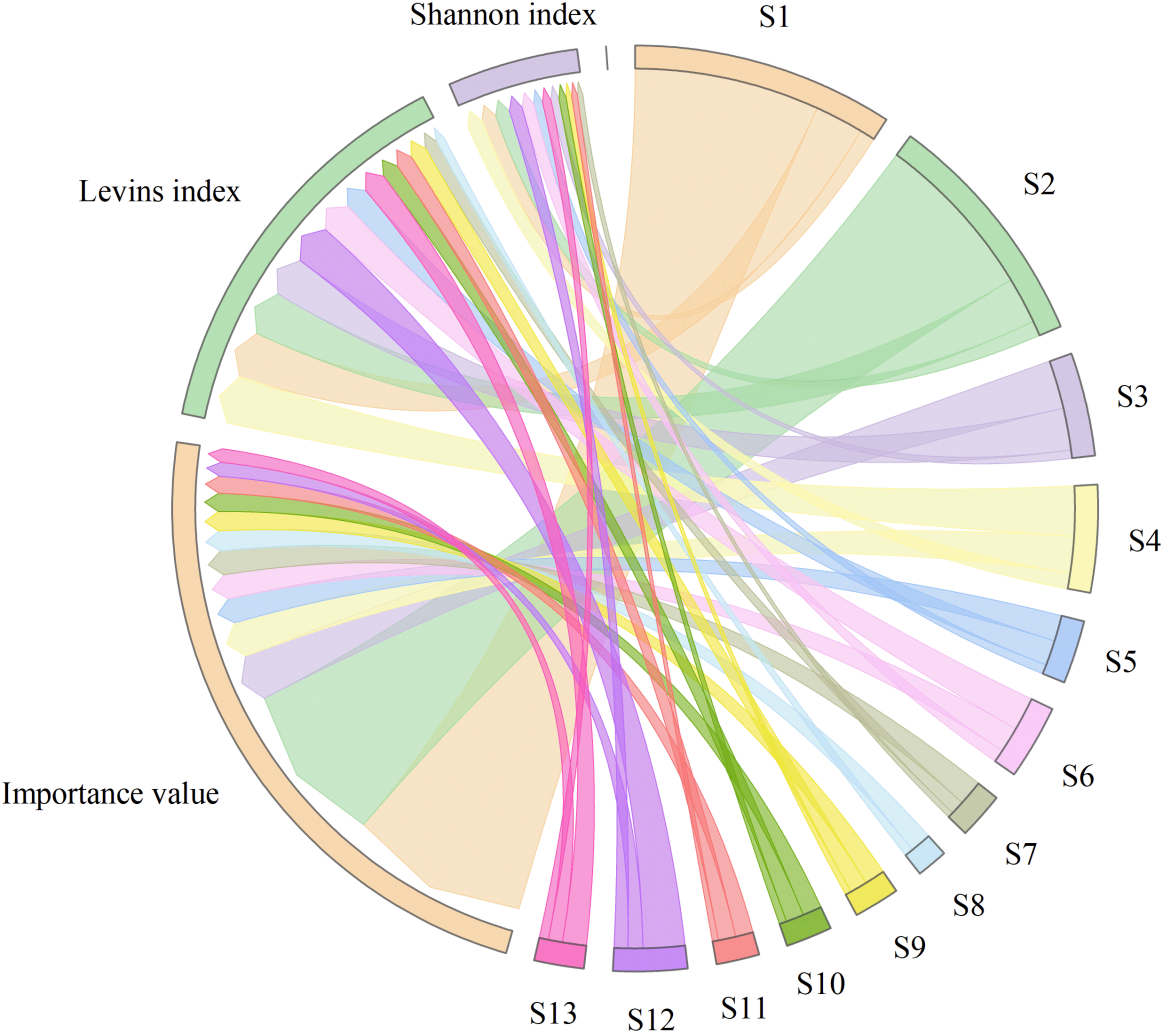
Importance value and niche breadth. S1-S13 represents different species, and the thickness of the bar in the circle represents the size of the corresponding indicator.

**Figure 2** shows the relationship between the niche breadth of dominant species and their importance values. As shown in **Figure 2**: *Castanopsis lamontii*, which has a lower importance value, exhibits a narrower niche breadth compared to *Castanopsis carlesii* and *Castanopsis eyrei*, both of which have higher importance values. A positive correlation exists between importance values and niche breadth; however, this relationship is not absolute. For some species, there is a notable discrepancy between the ranking of importance values and that of niche breadths. For instance, *Aidia cochinchinensis* has a relatively low importance value ranking but displays a comparatively high niche breadth among the studied species.

### 3.2 Niche overlap and niche similarity analysis

**Figure 3:**
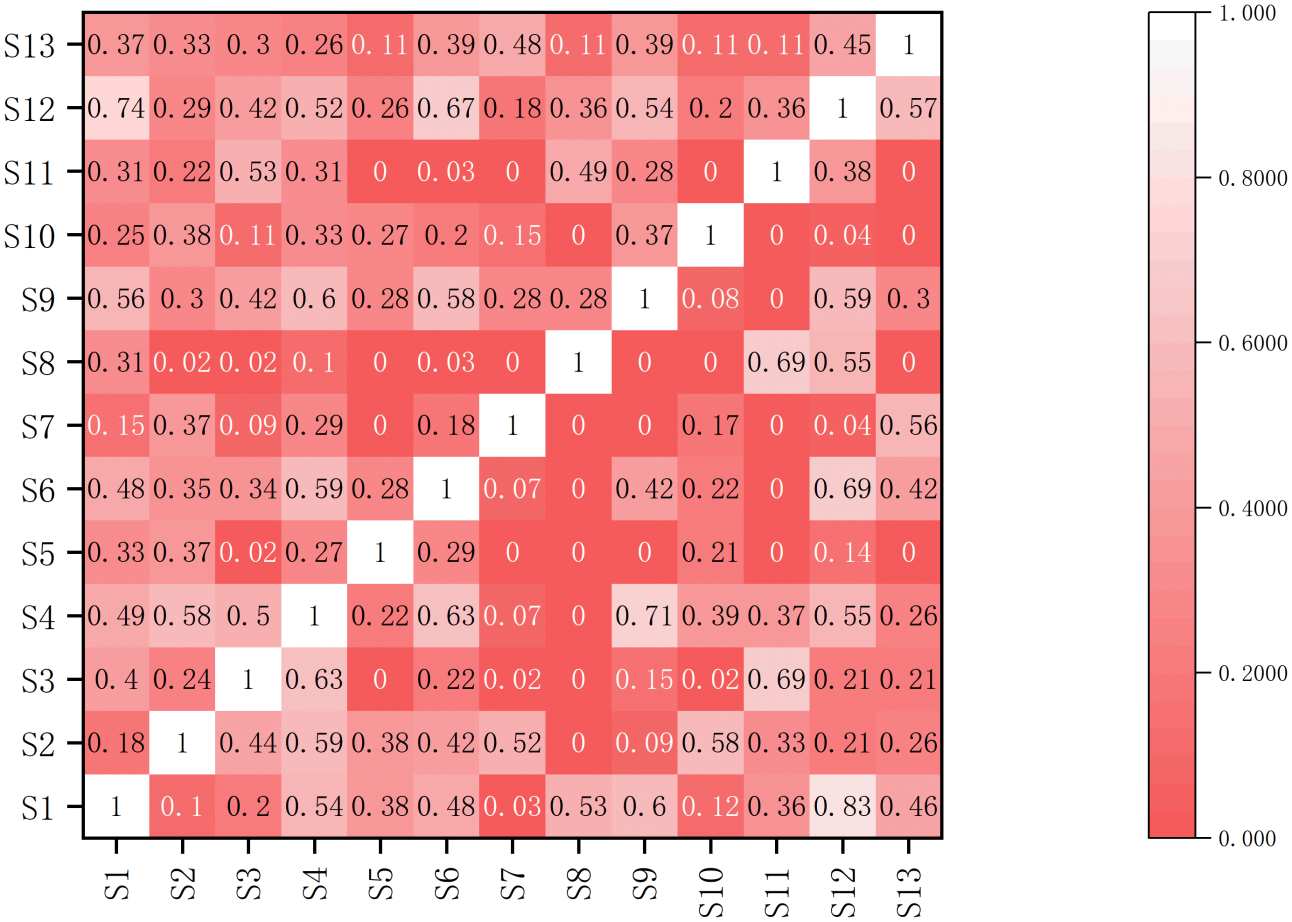
Pianka index and Schoener index of the dominant woody plants in the evergreen broad-leaved forest. Species numbers as in **Table 1**, the upper left part is the Pianka index and the lower right part is the Schoener index.

As shown in **Figure 3**, in the investigated plots, the Pianka index for 78 pairs of 13 tree species ranged from 0 to 0.83, while the Schoener index ranged from 0 to 0.74. The number of species pairs with Pianka and Schoener index values below 0.10 was 30 and 13 pairs, respectively, accounting for 38.46% and 16.67% of the total number of pairs. For instance, the niche overlap between *Myrsine seguinii* and *Castanopsis tibetana* is relatively low, approaching zero, indicating pronounced niche differentiation between these two species. The number of species pairs with Pianka index and Schoener index values within the interval [0.1, 0.5] was 30 and 55, respectively, accounting for 38.46% and 70.51% of the total number of pairs, and these species can capable of coexisting under resource-rich habitat conditions. Furthermore, there were 18 and 10 species pairs with Pianka index and Schoener index values greater than 0.5, accounting for 23.08% and 12.82% of the total number of pairs, respectively. Among them, the highest niche overlap was observed between *Castanopsis carlesii* and *Aidia cochinchinensis* (*O_ik_* = 0.83, *C_ik_* = 0.74), indicating significant convergence in their resource utilization patterns and supporting their stable coexistence in resource-abundant environments. The niche overlap of *Castanopsis carlesii* with other species is generally high, suggesting that *Castanopsis carlesii* shares numerous similarities with other species in resource acquisition.

### 3.3 Overall interspecies association

The overall interspecific association calculation for dominant woody plant species yielded a *VR* = 0.71 (<1), indicating negative interspecific associations among these dominant species in the evergreen broad-leaved forest. Based on the chi-square distribution table, when degrees of freedom (*df*) = 40, $\chi_{0.95,40}^{2}$=26.509, $\chi_{0.05,40}^{2}$=55.758, and $\chi_{0.95,40}^{2}$< W< $\chi_{0.05,40}^{2}$. The test statistic “*W*” (28.4) falls within the 90% confidence interval of the *χ*² distribution (26.509 < *W* < 55.758), meaning that the deviation of *VR* from 1 is not statistically significant, and the interspecific associations are insignificant. Consequently, the overall association among dominant tree species in the evergreen broad-leaved forest community of Meihua Mountain exhibits insignificant negative associations.

### 3.4 Chi-square test

**Figure 4:**
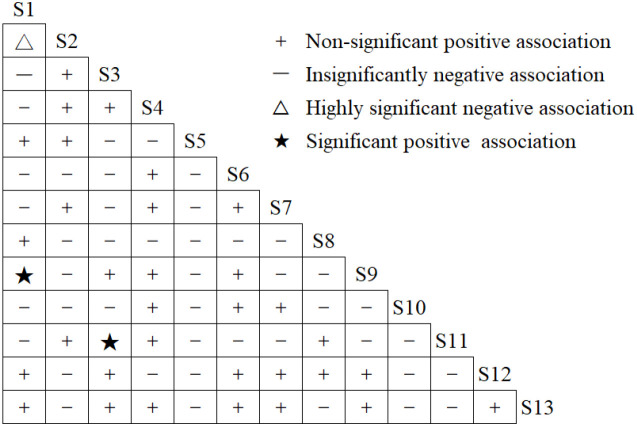
Semi-matrix diagram of *χ^2^* test for dominant species of woody plants. Species numbers as in **Table 1**.

**Figure 4** presents the *χ²* test results for interspecific associations among 78 dominant woody plant species pairs in evergreen broad-leaved forests of Meihua Mountain Nature Reserve, Fujian. The positive-to-negative association ratio was 0.77, with 34 pairs of positive associations (43.59% of the total pairs) and 44 pairs of negative associations (56.41% of the total pairs) among the 78 species pairs made up of dominant species of woody plants in evergreen broad-leaved forests in Meihua Mountain Nature Reserve, Fujian. Among these, 32 species pairs showed a non-significant positive association (*P* > 0.05), 2 species pairs exhibited a significant positive association (*P<* 0.05), 43 species pairs demonstrated a non-significant negative association (*P* > 0.05), and 1 species pair displayed a highly significant negative association (*P<* 0.01), which accounted for 41.03%, 2.56%, 55.13%, and 1.28% of the total number of pairs, respectively. These results indicate that negative interspecific associations prevailed in the community, with most species pairs (96.15%) showing non-significant associations (*P* > 0.05) suggesting independent species distributions.

### 3.5 Spearman rank correlation coefficient

**Figure 5:**
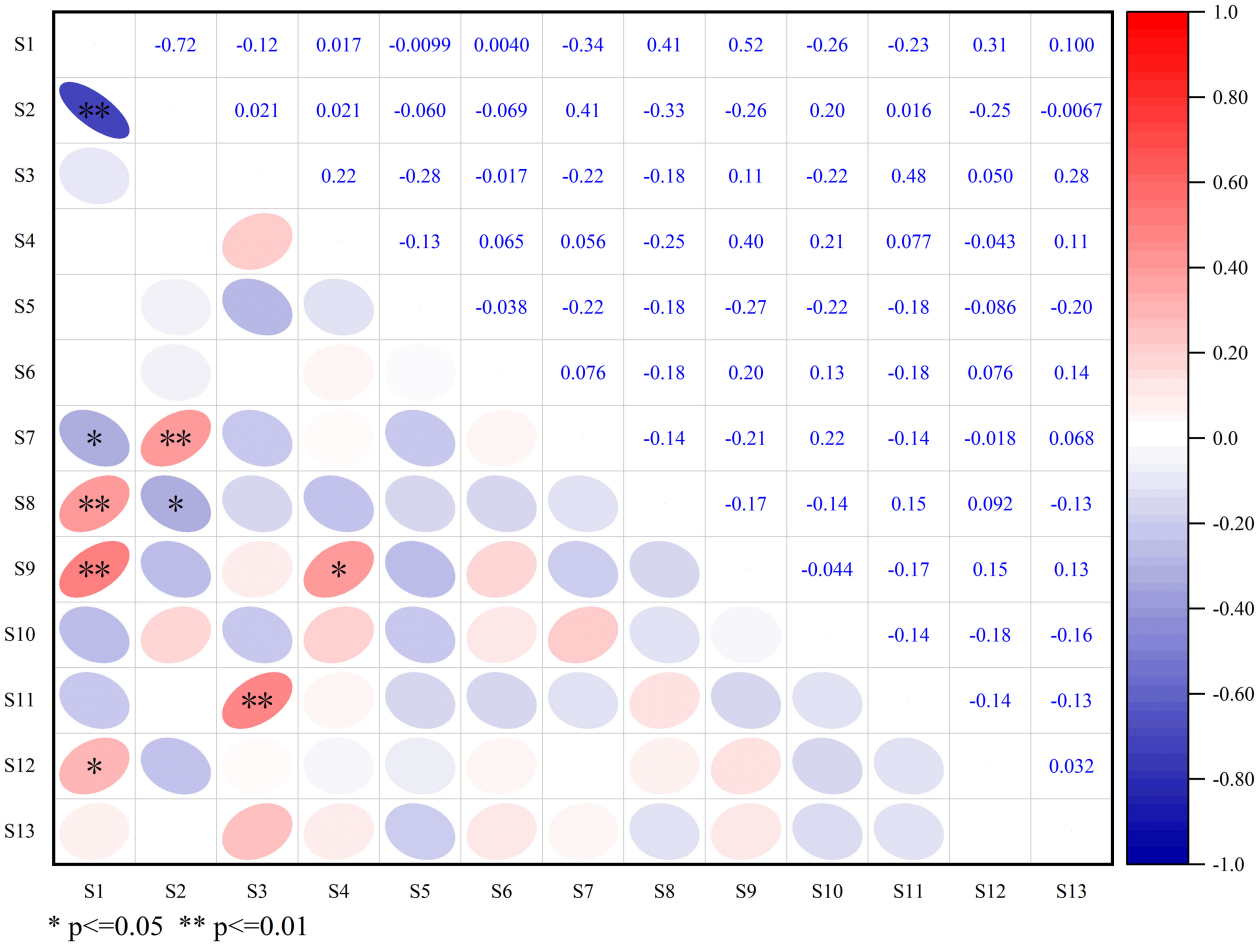
Spearman rank correlation coefficient of wood dominant species.

Spearman rank correlation analysis revealed 34 positively correlated species pairs and 44 negatively correlated pairs (**Figure 5**), yielding a positive-to-negative ratio of 0.77. Among these, four pairs showed highly significant positive correlations (*P* < 0.01), two exhibited significant positive correlations (*P* < 0.05). For instance, *Castanopsis carlesii* and *Sloanea sinensis* show a highly significant positive association. One displayed a highly significant negative correlation (*P* < 0.01), and four pairs demonstrated significant negative correlations (*P* < 0.05). Notably, non-significant negative correlation pairs (*P* > 0.05) constituted a substantial proportion of the total, consistent with the results of the chi-square test.

### 3.6 Regression analyses of interspecies association and niche overlap index

**Figure 6:**
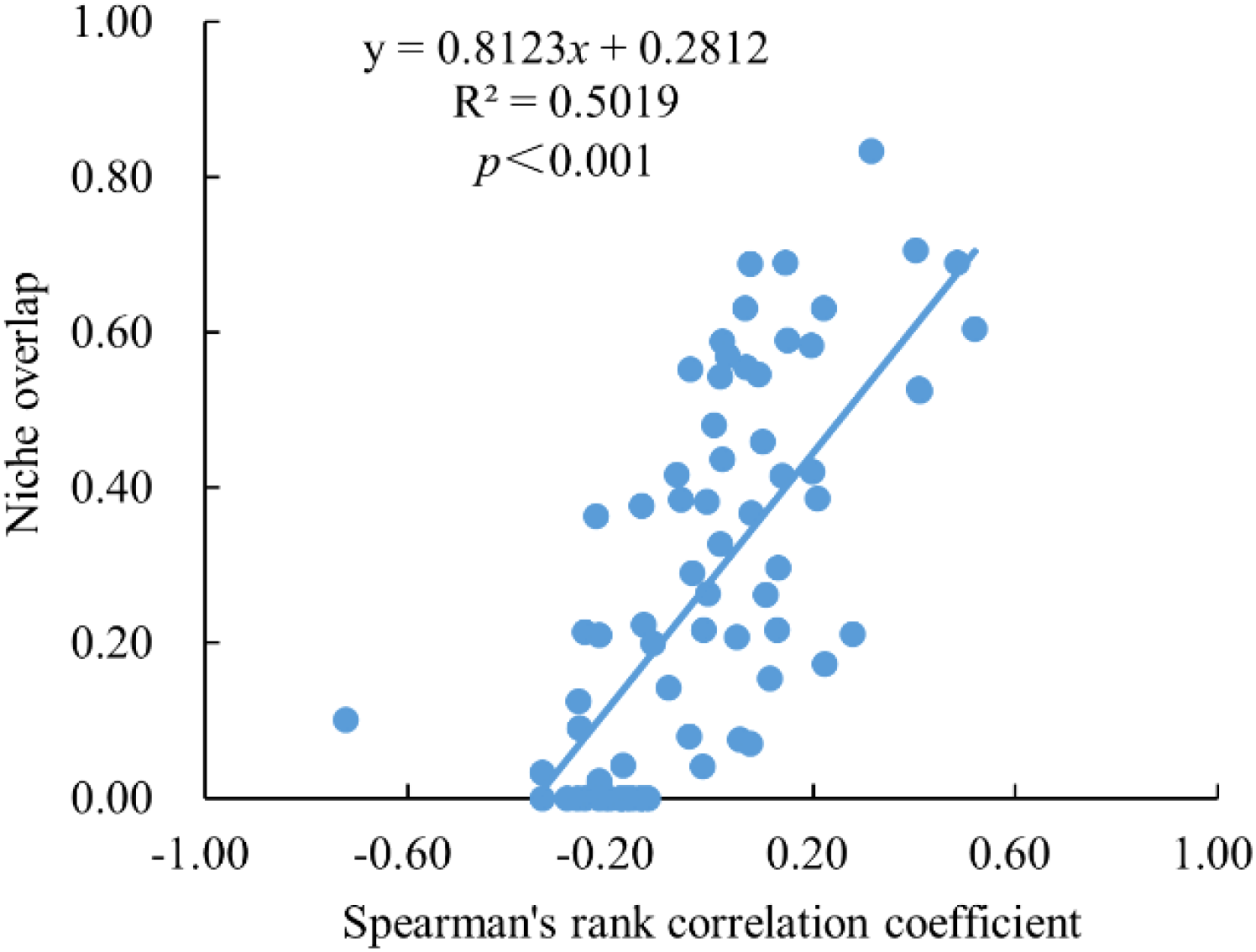
Regression analysis of interspecific association coefficients and niche overlap for dominant species.

**Figure 6** demonstrates highly significant positive correlations between niche overlaps Spearman rank correlation coefficients among dominant woody plant species in evergreen broad-leaved forests of Fujian’s Meihua Mountain Nature Reserve. The Spearman rank correlation coefficient (*R²* = 0.5019) showed a relatively good model fit. This result indicates that increased interspecific association among dominant species correlates with a higher degree of niche overlap.

## 4 Discussion

### 4.1 Ecological niche features of predominant woody plant species

Importance value serves as a key indicator for assessing a species’ status within a community and its ecological adaptability, with higher values reflecting greater ecological prominence (Granot and Belmaker, 2020). Additionally, niche breadth is a crucial measure of a species’ capacity to utilize d*iv*erse resources (Laaka-Lindberg et al., 2005). Analysis of the correlation between importance values and niche breadths revealed a general positive association, consistent with findings reported by Nan et al. (2023). In the communities at Meihua Mountain, dominant species such as *Castanopsis carlesii*, *Castanopsis eyrei*, *Phyllostachys edulis*, and *Machilus thunbergii*—characterized by high importance values—also exhibited broad niche breadths, indicating strong resource utilization capacity and environmental adaptability, enabling them to dominate the community. However, this positive relationship is not absolute, and interspecific variations exist. For example, while *Machilus thunbergii* possesses the widest niche breadth, *Castanopsis carlesii* has the highest importance value. This pattern aligns closely with the niche breadth rankings and significance levels for these two species observed in the community study by Ye et al. (2021). Field surveys and habitat analysis further revealed that many broad-leaved evergreen forests are distributed in mountain valleys. Specifically, *Castanopsis carlesii* was primarily found on sunny slopes and ridges within sample plots at elevations of 700–800 m. In contrast, *Machilus thunbergii*, typically a fast-growing small tree, is highly adaptable and commonly occurs in gullies and valleys, occupying a wider range of microhabitats within broad-leaved evergreen forest ecosystems, thereby maintaining a high niche breadth across heterogeneous environments. Furthermore, discrepancies in ranking were observed between *Rhododendron henryi* and *Aidia cochinchinensis*: *Aidia cochinchinensis* ranked twelfth in importance value but fifth in niche breadth, whereas *Rhododendron henryi* ranked fifth in importance value and seventh in niche breadth. The relatively high-water demand of *Rhododendron henryi* compared to other species may explain the mismatch between its importance and niche breadth (Feng et al., 2008). Survey data indicate that *Rhododendron henryi* is mainly concentrated in the understory at 500–700 m elevation on gentle slopes, where moisture loss is minimized, reflecting an uneven spatial distribution. Although *Aidia cochinchinensis* showed a relatively high frequency of occurrence in sample plots, its low individual abundance and smaller plant size contributed to its lower importance value. The observed inconsistency between importance values and niche breadth rankings suggests that importance value alone does not determine niche breadth; rather, species distribution frequency also plays a significant role, with more widely distributed species tending to exhibit greater niche breadth (Liu et al., 2023).

Niche overlap effectively quantifies the degree of similarity in resource use between species and reveals potential competitive interaction (Weber et al., 2023). Generally, when *O_ik_* value exceeds 0.6, species are considered to utilize resources in a highly similar manner (Wathne et al., 2000). In this study, six species pairs exhibited a niche overlap index greater than 0.6, indicating relatively high similarity in their resource requirements. Overall analysis revealed that 76.92% of the community’s species had a niche overlap index below 0.5, with an average index of 0.26 for the dominant species across the community. This indicates that most of the species showed relative independence in resource utilization and the competitive pressure between species was relatively small. However, compared to other evergreen broad-leaved forest communities—such as the Wuyi Mountain evergreen broad-leaved forest—niche overlap among species tends to be more prevalent and overlap values higher. This may be attributed to the coexistence of numerous species under similar environmental conditions, intensifying resource competition (Chen, 2016). Further analyses revealed that wide-niche species—*Castanopsis eyrei*, *Machilus thunbergii*, and *Castanopsis carlesii*—exhibited relatively high niche overlap and overall niche similarity with other species, whereas narrow-niche species such as *Myrsine seguinii* showed low Pianka and Schoener indices when paired with others. These findings indicate that species with broader niche breadths generally have greater access to resources, wider distributions, and higher niche overlap and similarity with co-occurring species (He et al., 2025). Nevertheless, niche overlap between species with large niche breadths can still be limited due to differences in specific habitat preferences and varying degrees of resource utilization, even under shared environmental conditions (Liu et al., 2022; Zheng et al., 2024). For instance, although *Castanopsis eyrei* and *Castanopsis carlesii* both possess broad niche breadths, their relatively low Pianka and Schoener indices suggest distinct ecological requirements and niche differentiation.

### 4.2 Characteristics of dominant interspecific linkages in woody plants

The successional trajectory of ecological communities can be reflected in the overall interspecific associations among species, which are closely linked to community stability (Su et al., 2015; Chen et al., 2023). During early developmental stages, interspecific associations tend to be unstable, with low or even negative correlations among species (Liu et al., 2017). As succession progresses, a stable climax community gradually emerges, characterized by increasing positive species associations and the stabilization of community structure and species assemblages (Gu et al., 2017). However, a study by Zhang et al. (2015) demonstrated that late-successional, old-growth broadleaf *Pinus koraiensis* forest communities also exhibited negative interspecific interactions, indicating that interspecific associations are not always positively correlated with community stability. In this study, dominant woody species in the old-growth broad-leaved evergreen forest community similarly showed non-significant negative overall interspecific associations and weak species interdependence. This pattern can be attributed to prolonged natural succession following the establishment of the Meihua Mountain Nature Reserve. Nevertheless, *Phyllostachys edulis* plantation cultivation remains a local economic activity. The evergreen broad-leaved forest communities adjacent to *Phyllostachys edulis* plantations are vulnerable to *Phyllostachys edulis* forest expansion and anthropogenic logging. These human-induced disturbances have not only disrupted understory vegetation structure but also significantly reduced biodiversity, resulting in a more pronounced secondary nature of the regional forest communities. Importantly, the ecological effects of anthropogenic disturbances vary substantially depending on their intensity. As shown in the study on *Pinus kesiya* by Jia et al. (2014), moderately disturbed communities exhibit distinct ecological characteristics: species richness is higher than in both undisturbed and severely disturbed communities, and these communities demonstrate notable advantages in stability, with stability indicators significantly surpassing those of the other two conditions.

It is commonly theorized that positively correlated species pairs reflect mutualistic or facilitative relationships, whereas negatively correlated pairs indicate antagonistic or competitive interactions (Schluter, 1984). The results of both the *χ*² test and Spearman rank correlation analyses in this study consistently revealed a greater number of negatively correlated species pairs than positively correlated ones, with most correlations being statistically non-significant. This suggests that dominant woody plant species exhibit spatial segregation, albeit with weak interaction intensity. Several factors may account for the prevalence of negative interspecific correlations. First, the Meihua Mountain Nature Reserve encompasses a complex topography with considerable elevational variation, leading to diverse microhabitats and differential disturbance regimes across sites—such as differences in elevation, slope aspect, and gradient—that shape distinct interspecific association patterns. Second, spatial heterogeneity, low niche similarity among dominant species, and niche partitioning contribute to non-significant negative associations. Variations in altitude and spatial distribution reduce resource overlap, minimizing direct competition. Third, plots at lower elevations have been subject to human-induced disturbances, which reduce species abundance and occurrence frequency, thereby weakening interspecific linkages. For example, *Castanopsis carlesii* and *Castanopsis eyrei* exhibit a highly significant negative correlation despite belonging to the same family (Fagaceae). Their divergent spatial distributions result in minimal resource use overlap and no direct competition, potentially explaining the observed negative association (Ren, 2020). Consistent with Sun et al. (1996), long-term microenvironmental adaptations may also drive such negative correlations. In contrast, highly significant positive correlations were detected among species with similar ecological requirements—for instance, *Castanopsis carlesii* and *Castanopsis tibetana* in evergreen broad-leaved forests. Similarly, *Phyllostachys edulis* and *Altingia gracilipes*, as well as *Castanopsis carlesii* and *Sloanea sinensis*, showed strong positive associations, likely due to convergent habitat preferences. These findings align with Yuan et al. (2023), demonstrated that species sharing similar habitat needs are more likely to form positive associations. Consequently, these positively associated species pairs tend to establish stable co-occurrence patterns within the community, playing a pivotal role in promoting ecosystem succession.

Furthermore, research on species’ ecological preferences and interspecific relationships has practical reference value for the restoration and reconstruction of community vegetation. In the context of vegetation restoration in the study area, it is advisable to prioritize tree species that exhibit strong positive associations with *Castanopsis carlesii*—such as *Sloanea sinensis* and *Castanopsis tibetana*—for co-planting in suitable habitats. This strategy enables more efficient utilization of limited resources, promotes mutual facilitation, and ultimately contributes to the establishment of a relatively stable forest ecosystem. During tending management of the *Castanopsis carlesii* community, it is recommended to moderately remove associated species such as *Myrsine seguinii*, which show negative or weak associations with *Castanopsis carlesii*. This practice can reduce interspecific competition and increase resource availability for the target species, thereby supporting sustainable forest development.

### 4.3 Linear regression analyses of ecological niches and interspecific associations

Interspecific association refers to the spatial co-occurrence patterns among different species within a community, reflecting the complex interdependence and mutual constraints between them. To some extent, niche overlap can reveal the underlying mechanisms driving interspecific associations, as a general positive correlation exists between the strength of species pair associations and their niche overlap indices. This study also detected significant positive correlations between niche overlap and Spearman rank correlation coefficients among dominant woody plant species in the Meihua Mountain Nature Reserve, Fujian Province. For instance, the species pair *Phyllostachys edulis* and *Altingia gracilipes* exhibited a Spearman correlation coefficient of 0.48 and a Pianka index of 0.69, indicating a mutually positive relationship. Moreover, positive associations were observed between *Castanopsis carlesii* and both *Castanopsis tibetana* and *Sloanea sinensis* in terms of both interspecific association and niche overlap. These findings are consistent with those of Xiao et al. (2024), who reported that stronger interspecific associations correspond to higher niche overlap. Concurrently, these results suggest that resource complementarity among species may serve as a mechanistic driver of positive interspecific associations.

## 5 Conclusion

Species diversity in the evergreen broad-leaved forests of Meihua Mountain Nature Reserve is high, with dominant woody species exhibiting niche differentiation and weak interspecific competition. The community currently shows non-significant negative interspecific associations and loose species interactions, suggesting unstable species relationships shaped primarily by human disturbance, environmental heterogeneity, and varying degrees of niche overlap. Reducing anthropogenic impacts and promoting coexistence among species with similar ecological requirements may facilitate stable community succession. This study is of critical importance for understanding community succession and species coexistence mechanisms, thereby contributing to ecosystem stability and biodiversity conservation. Based on these findings, precise population management can be implemented in forest conservation and management once species niche characteristics and symbiotic relationships are clarified. For example, applying thinning or selective cutting for species with high niche overlap can reduce the number of species pairs exhibiting negative interspecific correlations, thus promoting the stable development of forest ecosystems. Meanwhile, long-term monitoring based on *in-situ* conservation is recommended, focusing on species composition and interspecific ecological relationships to ensure mutualistic coexistence, enhance biodiversity, and improve community stability. Specific measures, such as establishing buffer zones or planting barrier tree species in areas where Phyllostachys edulis is expanding, can help mitigate its competitive dominance. This approach also provides a scientific basis for balancing ecological services with economic development. The study focuses on the interspecific relationships within the forest community. While it identifies human disturbance as a key factor influencing these relationships, the specific effects of varying disturbance intensities have not yet been clearly elucidated. Therefore, future research should prioritize investigating how different levels of anthropogenic disturbance shape interspecific associations and community dynamics.

## Data Availability

The original contributions presented in the study are included in the article/supplementary material. Further inquiries can be directed to the corresponding author/s.
